# 317. Invasive Fungal Infections in Critically-ill Patients with COVID-19 in Mexico City

**DOI:** 10.1093/ofid/ofab466.519

**Published:** 2021-12-04

**Authors:** Eduardo S Bojorges-Aguilar, Carla M Roman-Montes, Areli Martinez-Gamboa, Andrea Rangel-Cordero, Paulette G Diaz-Lomeli, Eduardo Rivero-Sigarroa, Jose Sifuentes-Osornio, Alfredo Ponce de Leon, Fernanda Gonzalez-Lara

**Affiliations:** Instituto Nacional de Ciencias Medicas y Nutricion Salvador Zubiran, Mexico City, Distrito Federal, Mexico

## Abstract

**Background:**

Invasive fungal infections (IFI) are emergent complications in SARS-CoV-2 infection. We aimed to describe the epidemiology, characteristics and outcome of IFI during the pandemic.

**Methods:**

Between March 2020 and April 2021, patients admitted to the Intensive Care Unit (ICU) in a COVID-19 center in Mexico City who developed IFI were included. COVID-19 associated pulmonary aspergillosis (CAPA) was defined according to the ECMM/ISHAM criteria. Demographic and clinical data were obtained from the electronic medical record. Descriptive analysis was made. The study was approved by the Institutional Review Board.

**Results:**

Sixty-seven (67/743, 9%) patients with COVID-19 developed IFI during ICU stay, of which 37 (55%) had CAPA, 24 (36%) had Invasive Candidiasis (IC), 3 Cryptococcosis and 3 pulmonary Mucormycosis. The median age was 57.5 (IQR 48-68) and 46 (69%) were male. Thirty-six (54%) had obesity and 20 (30%) type 2 diabetes. Sixty-two received COVID-19 directed therapy: 48/67 (72%) steroids, 4/67 (6%) tocilizumab and 8/67 (12%) were included in clinical trials. Among 24 patients with IC, 13 (54%) were fluconazole-resistant *C. parapsilosis*, 11 (46%) *C. albicans* and 2 *C. glabrata*. Twenty-two received antifungal treatment, 20 with echinocandins and 2 fluconazole. Among 37 CAPA, 8 (22%) were probable and 29 (78%) possible. Serum galactomannan was positive in 8 (22%), 33 respiratory cultures grew *Aspergillus* (31 tracheal aspirates and 2 bronchoalveolar lavage). *Aspergillus fumigatus* was the most frequent isolate in 18/33 (55%). Chest CT showed ground glass opacities in 21 (57%). Most received voriconazole (26/37, 70%). The median time from ICU admission to IFI was 9.5 (IQR 3-14) days. The median ICU and hospital stay length were 30 days (IQR 16-41) and 40 days (IQR 23-49), respectively. In-hospital mortality was 48%. The incidence rate of IC was higher early in the pandemic, due to Infection Control breaches, while higher CAPA incidence may have occurred later due to ventilation system gaps (Figure 1).

Bi-monthly Invasive Fungal Infection incidence rate/100 ICU admissions.

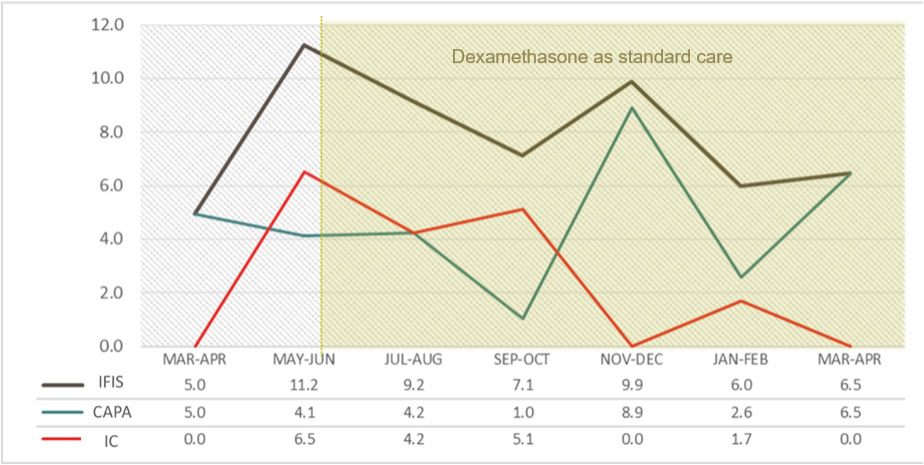

**Conclusion:**

We found 9% incidence of IFIs in critically-ill COVID-19 patients with high mortality. The majority received steroids, had obesity and had a prolonged hospital stay. Most had possible CAPA. An outbreak of fluconazole-resistant *C. parapsilosis* was found.

**Disclosures:**

**All Authors**: No reported disclosures

